# Twenty years of Prevention of Mother to Child HIV Transmission: research to implementation at a national referral hospital in Uganda

**DOI:** 10.4314/ahs.v22i2.5S

**Published:** 2022-08

**Authors:** Emily Namara-Lugolobi, Zikulah Namukwaya, Maxensia Owor, Joseph Ouma, Joyce Namale-Matovu, Clemensia Nakabiito, Christopher Ndugwa, Mary Glenn Fowler, Phillipa Musoke

**Affiliations:** 1 Makerere University-Johns Hopkins University Research Collaboration; 2 Makerere University Infectious Diseases Institute, Makerere College of Health Sciences; 3 Makerere University College of Health Sciences, Department of Paediatrics and Child Health

**Keywords:** PMTCT Research, PMTCT Program, sub-Saharan Africa

## Abstract

**Background:**

Over 90% of new paediatric HIV infections are acquired through mother to child transmission. Prevention of mother to child HIV transmission (PMTCT) research in sub-Saharan Africa informed WHO guidelines which enabled implementation of PMTCT programs globally.

**Objectives:**

To describe Makerere University-Johns Hopkins University (MU-JHU) perinatal HIV prevention research and implementation of the Mulago National Referral Hospital (MNRH) PMTCT program.

**Methods:**

Perinatal HIV prevention studies conducted at MU-JHU between 1997–2016 were summarized. Program aggregated data was extracted and analyzed using STATA 15.

**Results:**

In 1999, the HIVNET 012 study demonstrated that single-dose nevirapine (sdNVP) to the mother at onset of labor and to her newborn, reduced MTCT by nearly 50%. In 2016, the PROMISE study documented the safety and efficacy of ART during pregnancy and breastfeeding period. Program implementation at MNRH started in 2000. Uptake of HIV testing increased from 70% to 99% from 2006 onwards. sd NVP was the initial ARV regimen but by 2012, MOH recommended Option B+(triple therapy). MTCT rates reduced from 16.9% in 2001 to 2.3% in 2020.

**Conclusion:**

Perinatal HIV prevention clinical trials conducted at MU-JHU provided evidence to inform WHO PMTCT guidelines. MNRH program evaluation demonstrated the significant decline in MTCT rates over the last two decades.

## Background

### Epidemiology of HIV in Sub Saharan Africa

In sub-Saharan Africa, 75% of new HIV infections in individuals aged 15–19 years are among women and girls^1^. Ninety percent of children < 15 years of age are infected through mother to child HIV transmission (MTCT). Rates of MTCT have ranged from 25–45%, with the higher rates found in sub-Sahara Africa (SSA), where breast feeding is the norm. Increased access to antiretroviral therapy improved survival and quality of life for people living with HIV^2^. The current global ART coverage in pregnant women living with HIV is 85%^3^. Despite the increased ART coverage in pregnant women worldwide, an estimated 150,000 new pediatric infections occurred in 2020.

Uganda was severely impacted by the HIV pandemic with national seroprevalence rates peaking at 18% among adults; and 30% in pregnant women attending antenatal care in the late 1980s^4^. During that time, access to antiretroviral therapy for PLWA in SSA was inconceivable and elimination of mother to child transmission in resource limited settings unimaginable. Research conducted at Makerere University-Johns Hopkins University Research collaboration provided clinical evidence to inform policies which enabled implementation of PMTCT programs globally

With support from Global Fund for TB and Malaria and the Presidents Emergency Relief for AIDS Relief (PEP-FAR), countries in resource limited settings including Uganda scaled up access to ART treatment and PMTCT services. Coverage of ART during pregnancy and breastfeeding increased to > 95% by 2020 with two million paediatric infections averted since 2000(UNICEF) and a significant reduction in MTCT rates^5^,^6^.

The Makerere University-Johns Hopkins University Research collaboration (MU-JHU) was founded in 1988 by investigators from Makerere University and Case Western Reserve University, who subsequently moved to Johns Hopkins University. The late Prof. Francis Mmiro (consultant Obstetrician and Gynaecologist) and the late Prof. Christopher Ndugwa (consultant Paediatrician) from Makerere University and Prof. Brooks Jackson and Dr. Laura Guay from Case Western Reserve University were the founders of this important and long-lasting collaboration. They recognized the gap in knowledge about HIV in pregnant women and perinatally infected children. So, they set up a clinical research unit at the Mulago National Referral Hospital (MNRH) to conduct research for these unique and vulnerable populations. Initially, MU-JHU conducted observational studies to determine the natural history of HIV in preg nant women and their infants. Subsequently, clinical trials, to determine safety and efficacy of various antiretroviral drugs for PMTCT in resource limited settings were prioritized. The aim of this article was to review the perinatal HIV prevention clinical trials conducted at MU-JHU and to document the outcome of PMTCT program implementation at Mulago Hospital National Referral Hospital between 2000 and 2020.

### HIV Clinical Trials for Prevention of Mother to Child HIV Transmission

Since 1997, MU-JHU conducted several HIV clinical trials that informed policy on PMTCT in Uganda and worldwide. Most of this research was conducted through the National Institutes of Health, Division of AIDS networks; HIVNET, HPTN and IMPAACT Below is a chronological summary of the major PMTCT studies conducted at MU-JHU to date.

**1. HIVNET 012 (1997–1999):** HIVNET 012 was a randomized, controlled Phase III clinical trial designed to compare the safety and efficacy of short-course oral nevirapine (NVP) and zidovudine (ZDV) for preventing mother-to-child transmission of HIV-1 during labour and the first week of life. This landmark study conducted in Uganda, found that a short intrapartum/neonatal regimen of NVP given to the mother at the onset of labor and to the infant within 72 hours of life reduced the risk of perinatal HIV transmission among breastfeeding women in Uganda by 47% at 14–16 weeks and by 41% at 18 months compared to a short intrapartum/neonatal regimen of AZT^7^.

**Why was this study important?** A single dose of Nevirapine to the mother at labour onset and to the newborn lowered the risk of HIV-1 transmission during the first 14–16 weeks of life by nearly 50% in a breastfeeding population. The HIVNET 012 landmark trial showed that this simple, deliverable and inexpensive regimen could decrease mother-to-child HIV-1 transmission in less developed countries and informed WHO PMTCT 2001 guidelines.

**2. HIVIGLOB -SWEN study (2004–2006):** The SWEN study was a randomized controlled trial conduct ed in Uganda, Ethiopia and India to assess whether daily NVP given to breastfed infants through 6 weeks of age could decrease HIV transmission via breastfeeding. HIV infected women breastfeeding their infants were randomly assigned to either receive single dose nevirap ine (NVP 200mg to women in labor and NVP 2mg/kg to newborns after birth) or a 6 week extended dose of NVP (sdNVP plus NVP 5mg daily from days 8–42 for the infant). The study found that giving 6 weeks of daily NVP once a day until the baby was 6 weeks old lowered the risk of HIV transmission to the baby through breast feeding by 50%^8^.

**Why was this study Important?** This study informed the WHO policy that led to 6 weeks of NVP prophylaxis (extended NVP) to reduce HIV transmission in breastfeeding infants born to HIV infected women.

**3. HPTN 027 (2006–2007):** HPTN 027 was a random ized, double blind, placebo-controlled phase I trial to evaluate the safety and immunogenicity of an HIV vac cine (ALVAC-HIV vCP1521) in 60 infants born to HIV-1 infected mothers with CD4 counts > 500 cell/µL. The participants were randomized to the ALVAC vaccine or placebo. Infants were vaccinated at birth, 4, 8 and 12 weeks of age with ALVAC or placebo. Cellular and humoral immune responses were evaluated using IFN-γ ELISpot, CFSE proliferation, intracellular cytokine staining, binding and neutralizing antibody assays.

Reactogenicity and adverse events (AE) were graded using the 2004 DAIDS toxicity tables.

The study found that the HIV vaccine was well tolerated in HIV exposed infants with no severe or life-threatening reactogenicity events. Adverse events were equally distributed across both study arms^9^, ^10^.

**Why this study was important?** At the time of this study there was an urgent need for alternative interventions that provided protection from HIV infection to infants during breastfeeding as breastmilk continued to pose a risk of MTCT of HIV. This first pediatric HIV vaccine study showed that the ALVAC-HIV vCP1521 vaccination was feasible and safe in infants born to HIV-infected women in Uganda and that the conduct of high quality infant HIV vaccine trials was achievable in Africa.

**4. HPTN 046 (2008–2010):** This Phase III, randomized, placebo-controlled multicenter trial evaluated the efficacy and safety of NVP given to breastfeeding infants born to HIV infected mothers through 6 weeks of age compared to an extended regimen given through 6 months of age or cessation of breastfeeding, whichever occurred earliest.

The study found that the overall risk of HIV transmission through breast milk at age 6 months was lower with extended daily infant NVP, 1.1%, compared to 2.4% in infants in the placebo arm who had only 6 weeks of NVP (p=0.048). The study also demonstrated that extended infant NVP is most important for infants of mothers with high CD4+ cell counts (> 350 cells/mm^3^) who were not receiving antiretroviral therapy for their own health according to ART guidelines at the time; among these infants, breast milk transmission was much lower with 6 months of NVP, 0.7%, compared to 2.8% of infants in the placebo arm who received only 6 weeks of NVP (p=0.014).^11^

**Why was this study important?** Previous studies had shown that giving daily infant NVP for 6 weeks, 14 weeks or 6 months to breastfeeding infants reduces HIV transmission through breast milk compared to single dose NVP. However, no other study had directly compared 6 weeks of NVP to 6 months of NVP to determine if the longer regimen provides additional benefit for prevention of HIV transmission through breast milk. This study showed NVP prophylaxis can safely be used to provide protection from mother-to-child transmission of HIV-1 via breastfeeding for infants up to 6 months of age. This study informed the WHO revised infant feeding guidelines of 2010^12^.

**5. PROMISE (2011–2016):** The Promoting Maternal and Infant Survival Everywhere (PROMISE) multisite trial was a randomized open label strategy trial that compared the relative efficacy and safety of various proven antiretroviral strategies for the prevention of mother-to-child transmission during pregnancy and breastfeeding among asymptomatic HIV infected pregnant women with high CD4 counts not meeting treatment criteria.

In the antepartum component, women were randomly assigned to one of three regimens: zidovudine plus intra-partum single-dose NVP with 6 to 14 days of teno fovir and emtricitabine post-partum (zidovudine alone); zidovudine, lamivudine, and lopinavir – ritonavir (zidovudine-based ART); or tenofovir, emtricitabine, and lopinavir-ritonavir (tenofovir-based ART). All antepartum regimens were continued through 6 to 14 days post-partum. All infants received NVP from birth until postpartum randomization. The primary outcomes were HIV transmission at 1 week of age in the infant and maternal and infant safety. The study found that the rate of early HIV transmission was significantly lower in the arm with triple ART during pregnancy when compared to antepartum zidovudine alone followed by single dose NVP and 3TC/TDF 2 week tail at delivery (prophylaxis) (0.5% in the combined ART groups vs. 1.8%; difference, -1.3 percentage points; repeated confidence interval, -2.1 to -0.4)^13^.

**Why was this study important?** This study found that antenatal triple ART resulted in significantly lower rates of early HIV transmission than zidovudine prophylaxis during pregnancy; albeit with a significandy higher risk of adverse maternal and neonatal outcomes including low birth weight <2500 grams and/or preterm delivery < 37 weeks gestations. These findings of low rates of HIV transmission in the ART group supported the revised 2013 and 2015 WHO guidelines of Option B+ or univer sal ART to all pregnant women.

In addition to the above clinical trials, MU-JHU also conducted several implementation science studies that sought to explore and support breastfeeding attitudes and practices among HIV infected women following implementation of WHO infant feeding guidelines and improve service delivery in the PMTCT country programs. The findings of these studies are shown below.

**6. Use of peers, community lay persons and Village Health Team (VHT)** members improves six-week post natal clinic (PNC) follow-up and Early Infant HIV Diag nosis (EID) in urban and rural health units in Uganda: A one-year implementation study.

This study evaluated the use of HIV infected peer mothers (peers), community lay persons and Village health team (VHT) members to improve Post Natal Clinic (PNC) follow up and early infant diagnosis (EID) in urban and rural health units. HIV-infected women were recruited from three urban antenatal (Mulago, Rubaga and Mengo hospi tals) and one rural health centre (Mpigi health centre IV) between January and September 2010. The women were followed through delivery and the mother-infant pairs up to 14 weeks for EID. Peers, community lay persons and VHT members trained in basic PMTCT and reproductive health were assigned to the study clinic to support and follow study participants. Data at baseline (one year before the intervention) was compared with that during the one year study period among study participants.

The baseline six-week PNC follow up of mother infant pairs was 37.7 % and increased during the study period to 78.5 % an incremental difference of 39.4 % (P < 0.001). EID increased from a baseline of 53.6 % to 86.3 % during the study period, an incremental difference of 20.5 % (P < 0.001)^14^.

**Why was this study important?** This study showed that the use of peers improved early postnatal follow up and EID and should be implemented in other health units to support the PMTCT cascade.

**7. WHO 2010 infant feeding guidelines in re source-limited settings:** attitudes of human immuno deficiency virus-infected women and other role players in Kampala, Uganda.

The objective of this formative evaluation research conducted at Mulago Hospital, Kampala, Uganda from September to November 2011 was to describe the attitudes of human immunodeficiency virus (HIV)-infected women and other role players towards the World Health Organization (WHO) 2010 infant feeding guidelines.

Focus group discussions (FGDs) were held among five groups; including health workers and participants. The recommended introduction of complementary foods to infants at six months of age while their HIV-infected lactating mothers continued to breastfeed was supported by all of the health workers, but by only a minority of participants from each focus group discussion. The majority of FGD participants and the health workers were in favor of an HIV-infected lactating mother taking antiretroviral (ARV) drugs during the breastfeeding period, rather than the infant^15^.

**Why was this study important?** This study showed that while WHO infant feeding guidelines are supported by health workers, the population requires further engagement to improve exclusive breastfeeding knowledge, attitude and practices.

**Implementation of the PMTCT program at Mulago National Referral Hospital**

Following the release of the landmark HIVNET 012 study results in 1999, MU-JHU received Call to Action (CTA) funds from the United States Agency for International Development (USAID) through the Elizabeth Glaser Pediatric AIDS Foundation (EGPAF) to provide PMTCT services at the government-owned Mulago National Referral Hospital. The hospital was among the first five pilot sites to provide PMTCT services in Uganda. Program implementation commenced in 2000 and was implemented as a collaboration between Makerere University College of Health Sciences departments of Obstetrics and Gynaecology and the department of Paediatrics and Child Health and Makerere University – Johns Hopkins Research Collaboration (MU-JHU). The main goal of this program was to reduce the rate of Mother to Child Transmission (MTCT) of HIV among pregnant women attending the antenatal clinics at Mulago Hospital.

Initially, the package of intervention included; HIV counseling and testing, provision of NVP prophylaxis for mother and infant, health education on best infant feeding practices and early infant HIV diagnosis. These activities were conducted in the antenatal clinics, labour and delivery units and the postnatal clinic. In 2006, the program expanded services to include the PMTCT follow up clinic which provided comprehensive HIV/AIDS care including long-term care to HIV infected women, children and their partners. In 2016, the Department of Obstetrics and Gynecology Mulago National Refferal Hospital along with the PMTCT program shifted to a newly constructed Kawempe hospital that eventually became autonomous in 2018, as a National Referral Hospital. From 2006 to date, the program has been funded by the United States President's Emergency Plan For AIDS Relief (PEPFAR), through Centers for Disease Control (CDC).

### Data collection and analysis

PMTCT program client data was collected routinely using the Ministry of Health's Health Management Information System (HMIS) paper registers on antenatal attendance, hospital deliveries, PLHIV and HIV Exposed Infants and entered into an access database (2000–2016), later into Uganda EMR, a customized openMRS database (2017-date). In addition, aggregated data was collected as monthly reports and submitted to Uganda MoH; from 2011 onwards, this data was submitted to the DHIS 2 system, the national electronic database. The indicators collected changed over the years with the evolving guidelines and implementation strategies. For this review, we obtained aggregated data from both the paper records and the electronic registers. Patient level databases were reviewed and data extracted for indicators that were not routinely reported. Stata 15 was used for analysis^16^. The indicators that have been analyzed include first antenatal attendance, pregnant women who had a known HIV status at first ANC, HIV infected women initiated on ART, infant feeding practices and HIV exposed infants tested for the first DNA PCR. The prevalence of HIV was determined as a proportion of the total number of pregnant women newly diagnosed with HIV at first ANC plus women who reported for first ANC with a known HIV positive status over the total number of pregnant women with an established HIV status at first ANC. The proportion of women receiving ARVs was determined as the number of women who received any ARVs for PMTCT or for their health over the total number of HIV infected women identified at first ANC. Early Mother to Child Transmission of HIV was determined as the number of infants who tested positive at the first DNA PCR test over the total number of infants tested for first DNA PCR.

### Implementation of the PMTCT program at Mulago National Referral Hospital 2000–2020

**HIV Counselling and Testing**

The launch of PMTCT services in Uganda recommended HIV testing using the “opt-in” or voluntary counselling and testing approach (VCT) that encouraged pregnant women to test for HIV during pregnancy^17^. In 2002, Uganda released the first PMTCT policy guideline with more districts and health facilities on board, but maintained a VCT approach for HIV testing^18^.

The 2005 Uganda National guidelines for HCT^19^ changed implementation from VCT to Provider Initiated Counselling and Testing (PICT) in which all pregnant women attending ANC received routine counseling with “opt-out” for HIV testing.

**Figure 1:**
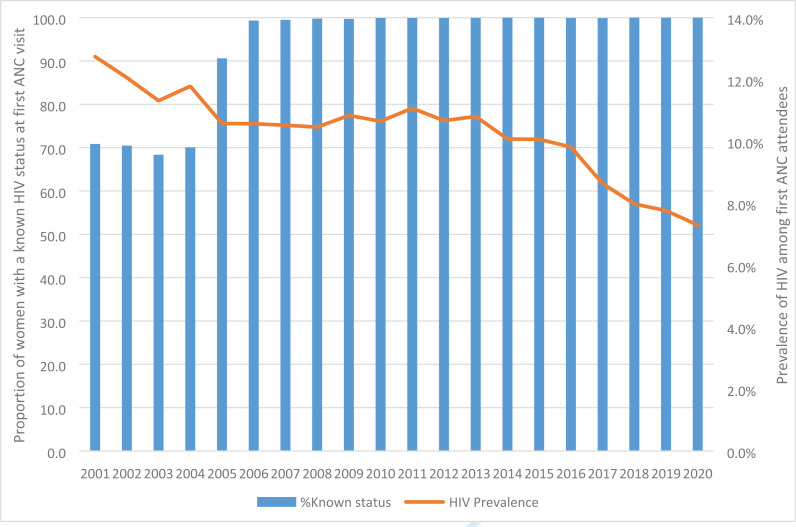
Proportion of Ugandan women attending antenatal care and testing for HIV infection 2001–2020

Between 2001 and 2004, the proportion of women tested for HIV at ANC remained low at an average of 70%, however, we documented a significant increase to 90.6% in 2005 following revision from the “opt-in” to “opt-out’ approach. This increase was sustained to 99.3% in 2006 and remained at close to 100% for the rest of the period up to 2020 (Figure 1). HIV prevalence was 12.1% in 2001 with an overall decline in the subsequent 4 years to 10.6%. It was then sustained at an average of 10.5% for the next 11 years. There was a steady decline from 9.8% in 2016 to 7.3% in 2020 following the adoption of the test-and-treat for all.

**Antiretroviral therapy in the context of PMTCT:** From 2000 to 2006, single dose NVP was provid ed to the mother during antenatal care, to be administered at the beginning of labor and to the infant after delivery^20^. However, following the launch of universal access to free ARVs in 2004 by the Ugandan government, recommending ART to adults with CD 4 count less than 200 cells/mm^3^ 21, a few women eligible for ART (CD4 count ≤ 200 cells/mm3 or WHO stage 3 and 4) reported to ANC already on ART. In 2006 the Uganda MOH adopted the latest WHO guidance^22^ that recommended combination therapy for PMTCT to women not eligible for ART. According to these guidelines, women with a CD4 count less than or equal to 350 cells/mm^3^ or who had a WHO stage III/IV were initiated on lifelong ART. Women not eligible for ART were initiated on zidovudine (AZT) at 28 weeks of amenorrhoea (WOA) or AZ-T/3TC at 36 WOA. At labour and delivery the women received sdNVP plus AZ-T/3TC to be taken for one week postpartum to avoid the development of NVP resistance. The guidelines further recommended sdNVP plus zidovudine syrup once daily for one week as infant prophylaxis. This was the prac tice from 2006 to 2010. In 2010, Uganda initially adopted OPTION A following updated guidance from WHO^23^,^24^. According to these guidelines, women with CD4 counts less or equal to 350 cells/mm^3^ received ART for life. However, pregnant women with CD4 counts greater than 350 cells/mm^3^ received AZT from 14 WOA, then sdNVP at labour and AZT/3TC for one week postpartum. The infants received NVP syrup from birth up to the end of the breastfeeding period. In 2012, Uganda opted for Option B plus^25^, ^26^. Therefore, all HIV infected pregnant and lactating women were initiated on ART irrespective of CD4 count or WHO stage. This test and treat strategy has been the main intervention for PMTCT implemented since 2012 to date.

**Figure 2:**
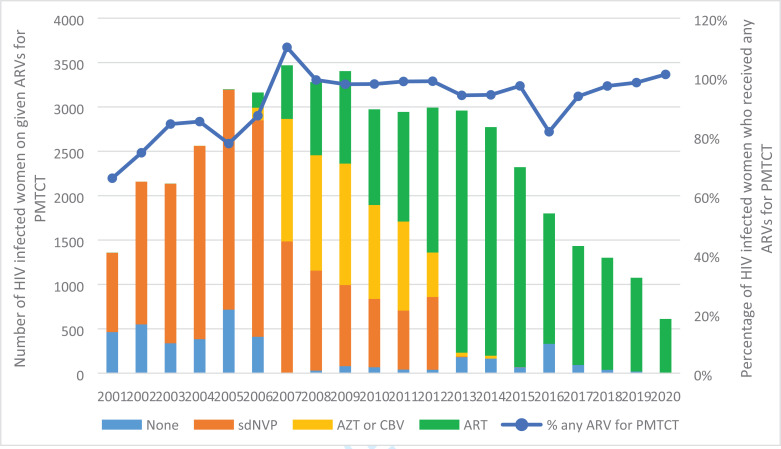
Proportion of Ugandan women who received ARVs during ANC (including those who were ART exposed)

From 2001 to 2006, the majority of the HIV positive women received sdNVP in ANC to be taken at onset of labaour (66% in 2001 to 77% in 2006), the rest of the women were not receiving ARVs (Figure 2). With the introduction of more efficacious regimens, the proportion of women receiving sdNVP drastically reduced from 85% in 2004 to 27% in 2012 and to zero for the remaining period up to 2020. With increased access to ART in 2006 for women with CD4<200cells/ml or stage III/IV disease, a few women received ART (5%) for their health in 2006 and a few received combination ARVs (4%). The proportion of women receiving ART increased steadily from zero percent in 2001 to 92% in 2013, and to close to 100% by 2020.

**Infant feeding practices among HIV infected women:** The feeding of infants and young children is crucial in determining the health, nutrition, survival, growth and development of the child. In the era of HIV, breastfeeding presents a dilemma given that it remains a potential route for mother to child HIV transmission accounting for 15% of all MTCT. The first recommendation for HIV positive pregnant women was replacement feeding, using infant formula provided by UNICEF^20^, ^27^. The second set of guidance was released in 2006 with, exclusive breastfeeding being recommended for at least 3–6 months. The third set of guidance from WHO was in November 2009 (Rapid Advice) and detailed guidelines came in July 2010 in Vienna^23^, ^25^. In the new 2010/2012 infant feeding guidelines, HIV positive mothers were strongly recommended to exclusively breastfeed until 6 months and then give complementary feeds from 6–12 months. However, if the mother preferred replacement feeding after counseling, she could do so if the AFASS (Acceptable, Feasible, Affordable, Sustainable and Safe) criteria was met. Infants confirmed HIV positive should breastfeed exclusively for 6 months, and then continue breastfeeding while adding complementary feeds until 24 months.

### Infant feeding practices for HIV Exposed Infants attending the PMTCT program at MNRH

During the initial PMTCT implementation, mothers living with HIV were encouraged to use replacement feeding. Figure 3 shows the choice of feeding for infants aged zero to three months seen over the years. Only (50%) of HEI exposed infants aged zero to three months were exclusively breastfeeding in 2001/2002. However, this proportion increased over the years in accordance with the changing infant feeding policies recommending exclusive breastfeeding for the first six months of life regardless of HIV status. From 2012 onwards, more than 90% of infants were breastfeeding exclusively for six months.

**Figure:**
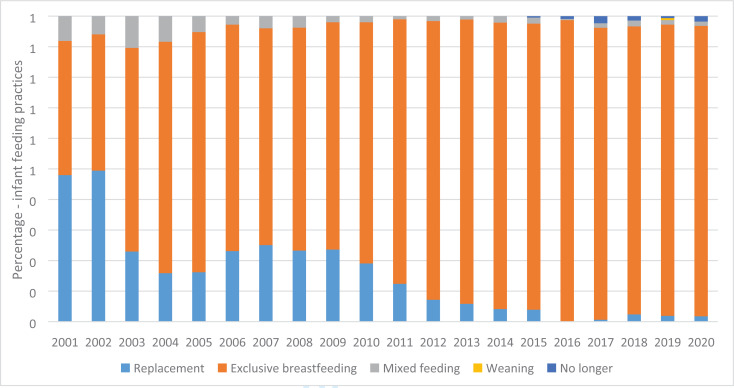


### Mother to Child Transmission rates at the first DNA PCR test

**Figure 4:**
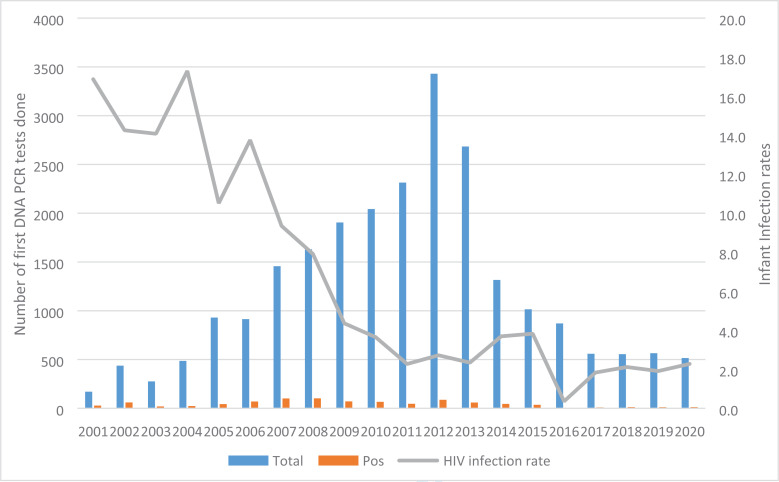
Number of first DNA PCR tests done and Uganda Mother to Child tra nsmission rates; 2001 – 2020

The total number of Ugandan infants screened for 1st HIV DNA PCR increased consistently from only 140 infants in 2001 to a highest number of 3430 infants in 2002 (Figure 4). This figure begun to reduce in 2013 and continued gradually for the next seven years to 576 in 2020. The infant infection rate reduced significantly from 16.9% in 2001, reaching 3.6% in 2010 (average decrease of 1.5% per year (95% CI -2.23 to -0.83, P<0.001). It remained fairly constant for the next 10 years and decreased to 2.3% in 2020 (average decrease of 0.07% (95% CI: -0.34 – 0.21, P=0.593). When compared to a MTCT rate of 36% without any intervention, this program evaluation shows averted infant infections ranging between 59.3% in 2001 to 93.5% in 2020.

## Discussion

Over the last 20 years, MU-JHU conducted multiple cutting edge perinatal HIV prevention clinical trials which demonstrated the efficacy of various antiretroviral regimens for PMTCT. The first study HIVNET012, a landmark trial demonstrated the efficacy of sd NVP to mother and her newborn, and provided proof of concept that a safe and efficacious regimen could be successfully implemented; enabling the initiation of PMTCT programs in resource limited settings globally^28^. Subsequently, other studies reported the benefits of extended infant NVP prophylax is during breastfeeding in reducing breastmilk transmission^8^, ^11^, ^29^. Building on earlier clinical trials, in 2016, the PROMISE study confirmed the safety and efficacy of triple antiretroviral therapy in pregnant and breastfeeding women to further reduce MTCT which supported the 2013 WHO guidelines for the elimination of mother to child HIV transmission (Option B+)^13^.

At the MNRH, mother-to-child HIV transmission rates significantly reduced over the 20 years of PMTCT program implementation; from 16.9% in 2001 to 2.3% in 2020, at this tertiary hospital in Uganda. The MTCT rates varied according to the coverage of ARVs for PMTCT and the effectiveness of the regimens in place during the different time periods. Be tween 2001 and 2006, sdNVP was the main regimen used for PMTCT, the uptake of ARVs among HIV infected women was sub-optimal ranging from 66% in 2001 to 87% in 2006 corresponding to MTCT rates between 16.9 to 13.8%. Compared to the MTCT rate of 35%^30^ among breastfeeding populations without any intervention, this rate represented a significant reduction among HIV exposed infants and was comparable to other studies^31^, ^32^ and program evaluations^33^. The introduction of combination ARVs for PMTCT in 2006 coupled with increased ARV coverage among HIV infected pregnant women corresponded to a period of further decline in MTCT rates from 9.4% in 2007 to 3.6% in 2010 which was sustained after the introduction of Option B plus.

The results of HIV testing during antenatal care showed the impact of change in policies on the uptake of HIV testing in ANC. Between 2001 and 2005, when Voluntary Counselling and Testing (VCT) was the recommended model for providing HIV testing, only 70% of new ANC attendees on average had a known HIV status. This increased to 90% in 2016 and over 99% in 2017 after policy change to Provider Initiated Counseling and Testing (PICT) and was maintained and in keeping with other studies in Uganda and sub Saharan Africa^17^, ^33^. The prevalence of HIV in ANC has remained fairly constant at an average of 10% between 2000 to 2016 and slightly decreasing thereafter to 7.3% in 2020. This is much lower than that reported in Southern African countries^34^, but close to the national prevalence of HIV among women at 7.6% reported in 2018^35^.

This PMTCT program evaluation further highlighted the changing infant feeding practices corresponding to the rapidly changing WHO guidelines. Despite the initial guidelines recommending replacement feeding and formula being provided on site, only 50% of infants received replacement feeds in 2001 and 2002. However, this significandy decreased over the years, when follow on infant feeding guidelines recommended exclusive breastfeeding^12^, ^36^. Close to 10% were providing mixed feeds, a practice that was associated with increased risk of MTCT. The poor uptake of formula milk by postpartum women may have been related to the stigma of not breastfeeding and its association with HIV^37^. In contrast, uptake of formula in Botswana was more acceptable; but unfortunately, among the many infants who were formula fed, there was an increased risk of diarrhea, malnutrition and death due to contamination in the ground water^38^. Similar findings of increased diarrhea and malnutrition in HIV exposed uninfected infants who were not breastfed were reported from other SSA countries. In 2010, WHO revised the infant feeding guidelines to recommend exclusive breastfeeding for infants below 6 months of age in resource limited settings, regardless of maternal HIV status^12^.

Given that aggregated data was used for this program evaluation, the predisposing factors to MTCT of HIV could not be studied. The presence of a high viral load has been highlighted as the single most important factor associated with the likelihood of MTCT of HIV^39^–^41^ irrespective of infant feeding preferences. Determinants of high viral load include recent infection, duration on ART and adherence to ARVs all compounded with cultural and social economic barriers including sexual and gender based violence.

## Limitations

Being hospital based, the results may not be representative of the general population. Other challenges inherent with program data include incompleteness and data accuracy. In addition, unlike longitudinal data, the use of aggregated data can only be used as a proxy because outcomes are not linked to the individual participants that were exposed to an intervention. Nevertheless, this evaluation was important given that it demonstrated the effectiveness of PMTCT implementation in a busy tertiary institution over two decades and these results are comparable to those reported in other programs in Sub Saharan Africa.

## Conclusion

This program evaluation of PMTCT at a high volume national referral hospital shows the sustained impact of quick adoption of evidence-based policies on prevention of mother to child transmission in a resource limited setting over the last two decades. To achieve elimination status by 2025, there is need to further adopt and implement to scale proven primary prevention methods including oral and long acting injectable PREP, the Dapivarine ring; and to find innovative ways to retain pregnant and breastfeeding mothers living with HIV in care and on life-long treatment.
